# Reconsidering the Widespread Use of Active Vitamin D Analogues for Osteoporosis in Japan: A Call for Evidence-Based Prescription Practices

**DOI:** 10.31662/jmaj.2025-0401

**Published:** 2025-11-28

**Authors:** Osamu Uemura

**Affiliations:** 1 Department of Pediatrics, Ichinomiya Medical Treatment & Habilitation Center, Ichinomiya-City Aichi, Japan

**Keywords:** active vitamin D analogues, osteoporosis, rickets, Sclerostin, kidney impairment, ectopic calcification

## Abstract

In Japan, active vitamin D has been widely prescribed for osteoporosis due to its regulatory approval, in contrast to international practice where it is not considered standard therapy. However, growing evidence indicates that these agents offer limited efficacy in fracture prevention and may pose significant risks. Mechanistically, active vitamin D increases both serum calcium and phosphate, which, in the absence of bone-forming signals such as growth, fracture healing, or high mechanical loading, may promote ectopic calcification rather than bone strengthening. Osteoporosis, particularly in low-turnover states common in aging or immobilization, is poorly responsive to active vitamin D, as calcium incorporation into bone is physiologically limited. Clinical trials have shown little benefit of vitamin D supplementation in individuals without deficiency, and adverse outcomes such as hypercalcemia, nephrolithiasis, kidney impairment, and vascular calcification have been documented, including a recent case series in patients with severe motor and intellectual disabilities. Despite these concerns, Japanese guidelines continue to list active vitamin D as an effective option, diverging from the recommendations of the European Society for Clinical and Economic Aspects of Osteoporosis and the United States Endocrine Society, which reflect nuanced, evidence-graded recommendations rather than explicit discouragement in primary osteoporosis. The persistence of widespread prescribing in Japan likely reflects historical practices, regulatory frameworks, and economic incentives. Given the aging population and high prevalence of osteoporosis, reconsideration of this approach is warranted. Prescribing should be restricted to clear endocrine indications such as hypoparathyroidism or chronic kidney disease-related secondary hyperparathyroidism. Aligning national practice with international standards, while promoting nutritional vitamin D and other evidence-based therapies, could reduce unnecessary harm and improve healthcare efficiency.

## Introduction

In Japan, active vitamin D has long been used as standard therapy for osteoporosis, partly due to regulatory approval for osteoporosis, rickets, and osteomalacia. Regulatory approval allows physicians to prescribe and obtain insurance reimbursement for these agents, but this does not necessarily mean that they are recommended by evidence-based clinical guidelines, which prioritize therapies with proven fracture-reduction benefit. This is in contrast to most countries, where such indications are absent. This unique regulatory stance has encouraged routine prescriptions even in contexts where evidence for benefit is weak or absent. As global guidelines increasingly recommend against the routine use of active vitamin D for primary osteoporosis management because of questionable efficacy and potential safety concerns, it is timely to reflect on whether Japan’s approach should evolve toward closer alignment with international standards. Consideration should be given to whether continued widespread use is justified, in light of both the potential for harm and the availability of alternative, evidence-based strategies.

## Mechanistic Considerations

An important determinant of whether bone is in a ‘calcium-demanding’ state is the activity of sclerostin, a glycoprotein predominantly secreted by osteocytes. Sclerostin inhibits the Wingless/Integrated (Wnt)/β-catenin signaling pathway, thereby suppressing osteoblast differentiation and activity. In situations where mechanical loading is low or systemic signals do not favor bone formation―such as prolonged immobilization, aging, or microgravity―sclerostin levels increase, leading to suppression of bone formation despite adequate or even elevated serum calcium and phosphate. Conversely, conditions that stimulate bone formation, including growth, fracture healing, and high-impact physical activity, reduce sclerostin expression, enhancing osteoblastic bone formation and mineral incorporation.

This regulatory mechanism is crucial in understanding the potential harm of active vitamin D administration in osteoporosis. When sclerostin levels are high, the skeleton is not primed to utilize additional calcium for bone formation; instead, increased serum calcium and phosphate levels―augmented by active vitamin D―predispose to ectopic calcification. Thus, evaluating sclerostin-related signaling provides a more accurate physiological framework for deciding whether calcium supplementation or active vitamin D therapy is likely to benefit bone health or merely increase systemic calcification risk.

Additional physiological context: Vitamin D’s principal role is to maintain serum calcium concentration by promoting intestinal absorption and kidney reabsorption of calcium, thus indirectly supporting bone mineralization. However, in calcium-deficient states, active vitamin D (1,25-(OH)_2_D) synthesis is upregulated along with parathyroid hormone (PTH), leading to bone resorption as a direct effect to restore calcium homeostasis. This means that in such situations, vitamin D can actually lower bone mass. Active vitamin D is the only endogenous factor that increases both serum calcium and phosphate simultaneously; when bone formation is not physiologically demanded, such an increase favors ectopic calcification in soft tissues, including the vasculature, myocardium, and kidneys. The decision of the body to form bone is governed by signals such as mechanical loading, growth, and hormonal cues (e.g., growth hormone, sex hormones). Without such cues, elevated calcium and phosphate result primarily in extra-skeletal calcification.

Osteoporosis is characterized by reduced bone mass and impaired microarchitecture, with bone remodeling often in a low-turnover state. Active vitamin D increases intestinal calcium absorption but does not directly stimulate bone formation. In low-turnover osteoporosis, calcium incorporation into bone is physiologically limited. Exogenous active vitamin D may elevate serum calcium and phosphate, increasing the calcium-phosphate product (Ca×P) and potentially promoting ectopic calcification. Importantly, Ca×P lacks a central homeostatic control mechanism, being regulated independently by hormones such as PTH and fibroblast growth factor 23 (FGF23).

## Clinical Evidence on Efficacy

Nutritional vitamin D plays an essential role in maintaining serum 25(OH)D levels, and supplementation is recommended for individuals with deficiency. Compared with active vitamin D, nutritional vitamin D is essential for maintaining adequate 25(OH)D levels and, in individuals with deficiency, may reduce fracture risk. However, randomized controlled trials have shown that high-dose nutritional vitamin D supplementation fails to improve bone strength or volumetric bone mineral density in individuals without vitamin D deficiency ^(1)^. Importantly, supplementation does not significantly increase the Ca×P, making it safer than active forms. The role of 1α,25-dihydroxyvitamin D appears primarily to maintain calcium homeostasis during periods of active bone remodeling, rather than to promote bone accrual in a quiescent skeleton. In Japan, despite limited fracture prevention evidence, active forms remain widely prescribed.

## Clinical Evidence on Risks

In clinical practice, serum calcium should be measured before and periodically during active vitamin D therapy, as inadequate monitoring can delay recognition of hypercalcemia and its complications. Risks associated with active vitamin D include hypercalcemia, nephrolithiasis, kidney impairment, and vascular calcification. Our recent case series documented patients with severe motor and intellectual disabilities who developed kidney dysfunction during prolonged alfacalcidol therapy; kidney function improved upon drug withdrawal ^(2)^. Such cases highlight potentially preventable harm from inappropriate prescribing.

In addition, according to the official interview form for Edirol (eldecalcitol), serious adverse reactions include hypercalcemia (1.5%) and urolithiasis (0.9%). Laboratory abnormalities were also common: increases in serum calcium (15.0%) and urinary calcium (20.3%) were observed in pooled clinical trial data ^(3)^.

## International Guidelines versus Japan

The Japanese Osteoporosis Society’s 2023 guidelines list active vitamin D as effective treatments for osteoporosis. In contrast, the European Society for Clinical and Economic Aspects of Osteoporosis and the United States Endocrine Society recommend against its routine use in primary osteoporosis, citing limited efficacy and risk of adverse effects ^(4), (5)^. These differences reflect divergent interpretations of the evidence base and varying thresholds for acceptable risk. The omission of active vitamin D should be understood as prioritizing therapies with strong fracture-reduction evidence rather than prohibiting its use.

## Discussion

From a mechanistic and physiological perspective, osteoporosis and vitamin D deficiency are distinct entities. Osteoporosis often represents an adaptive reduction in bone mass due to low mechanical loading (e.g., in bedbound patients with severe motor and intellectual disability [SMID] or swimmers), whereas vitamin D deficiency primarily impairs bone mineralization. In low-turnover osteoporosis, increasing serum calcium and phosphate through active vitamin D administration does not stimulate bone formation; rather, it predisposes to calcium-phosphate deposition in non-skeletal sites. This is consistent with clinical experience in SMID and other low-loading states, where vitamin D supplementation fails to improve bone strength but increases the risk of ectopic calcification. Preventive strategies should consider whether the patient is in a “bone-forming mode”―such as during growth, fracture healing, or periods of significant mechanical loading―before attempting to increase mineral availability. In the absence of these conditions, especially in older adults with low bone turnover, active vitamin D may shift the balance toward harm rather than benefit.

The persistence of active vitamin D prescribing in Japan may be rooted in historical practice patterns, regulatory approval, and clinician familiarity. Economic factors, including pharmaceutical marketing and insurance reimbursement structures, may also play a role. Given the aging population and high prevalence of osteoporosis, even small increases in drug-related harm can have substantial public health implications. Revisiting prescribing practices with reference to international recommendations could support patient safety and healthcare efficiency. A risk-stratified approach is recommended: reserve active vitamin D for hypoparathyroidism, chronic kidney disease (CKD)-mineral and bone disorder, or severe malabsorption with regular calcium monitoring, and use bisphosphonates or denosumab as first-line therapy for high-risk osteoporosis.

### Recommendations

1. Reassess the approved indications for active vitamin D in Japan.

2. Update national osteoporosis guidelines to limit active vitamin D use to specific endocrine disorders (e.g., hypoparathyroidism, CKD-related secondary hyperparathyroidism).

3. Promote nutritional vitamin D and other evidence-based osteoporosis treatments as first-line options.

4. Increase clinician and public awareness of the risks associated with active vitamin D.

## Conclusion

Japan’s widespread use of active vitamin D for osteoporosis is inconsistent with international evidence-based practice. Given the limited efficacy and potential risks, prescribing should be restricted to clearly defined indications. Considering greater alignment of Japanese practice with global standards could support patient safety and optimize resource utilization.

## Article Information

### Author Contributions

Osamu Uemura: The author solely contributed to the conception, design, interpretation, and writing of this manuscript.

### Conflicts of Interest

None
